# Self-efficacy moderates the relationship between health comparisons and social exclusion: results of the German ageing survey

**DOI:** 10.1186/s12955-017-0831-5

**Published:** 2017-12-29

**Authors:** André Hajek, Hans-Helmut König

**Affiliations:** Department of Health Economics and Health Services Research, Hamburg Center for Health Economics, University Medical Center Hamburg-Eppendorf, Hamburg, Germany

**Keywords:** Health comparisons, Social exclusion, Self-efficacy, Older adults, Asymmetric effects

## Abstract

**Background:**

Little is known about the consequences of health comparisons. Negative health comparisons might, for example, result in emotions such as anger or frustration. These negative emotions might intensify feelings of social exclusion. Thus, the objective of the current study was to investigate whether health comparisons are associated with social exclusion. Moreover, it was examined whether the relation between health comparisons and social exclusion is moderated by self-efficacy.

**Methods:**

We analyzed cross-sectional data of *N* = 7838 individuals from the German Ageing Survey. The German Ageing Survey is a representative sample of community-residing individuals aged 40 and over. An established social exclusion scale was used. The degree of self-efficacy was measured according to Schwarzer and Jerusalem. Health comparisons were measured with the question “How would you rate your health compared with other people your age” (Much better; somewhat better; the same; somewhat worse, much worse).

**Results:**

Multiple linear regressions revealed that negative health comparisons were associated with feelings of social exclusion in men, but not women. Furthermore, positive health comparisons were weakly associated with decreased feelings of social exclusion in men. The association between negative as well as positive health comparisons and social exclusion in men was significantly moderated by self-efficacy.

**Conclusions:**

The findings of the present study suggests that negative health comparisons are associated with feelings of social exclusion in men. In conclusion, comparison effects are not symmetric and predominantly upwards among men in the second half of life. Strengthening self-efficacy might be fruitful for attenuating this relationship.

## Background

Previous studies have shown that income increases over time do not increase subjective well-being (SWB) in the long term [1]. However, individuals that are more affluent are more satisfied cross-sectionally [1]. This phenomenon is widely known as “Easterlin-Paradox” [1]. A possible explanation might be that relative income matters much. A substantial body of evidence exists demonstrating that the income in comparison to other peer or reference groups such as workmates is strongly associated with SWB [2–4]. When the income is lower compared to the income of a reference group, individuals report lower SWB. On the other side, when the income is higher compared to the income of a reference group, SWB is not increased, concluding that income comparisons are usually upwards. Another study has suggested that bad events are stronger than good ones [5]. It has often been found that the effects of upwards comparisons are more pronounced in men [4, 6].

Most of these studies focused on income comparisons. However, two recently published studies [7, 8] have proposed that the concept of comparisons is a broad one and might be extended to the health domain. Using the question “how would you rate your health compared with other people your age” (Much better; somewhat better; the same; somewhat worse, much worse) these studies have shown that negative health comparisons (“somewhat worse” or “much worse”) are associated with a decreased SWB [7] and negative health outcomes [8] (e.g., functional decline or more depressive symptoms). However, it remains an open question whether health comparisons are associated with social exclusion. To put it differently: Studies are missing focusing on the relationship between health comparisons and social exclusion. Socially excluded individuals are “people not being involved in different areas of life and their community, e.g. being unemployed and having a limited social network” [9]. This area of research is important because social exclusion leads to, e.g., (unintentionally) self-defeating behavior [10] and even suicide [11].

Negative health comparisons might, for example, result in emotions such as anger or frustration [12, 13]. These negative emotions might intensify feelings of social exclusion. In sum and in accordance with the concept of asymmetric comparisons, we hypothesize that negative health comparisons are associated with more pronounced feelings of social exclusion. Furthermore, we hypothesize that the negative health comparisons are particularly important for men because of their competitive characteristics [14] that might lead to feelings of inferiority and feelings of social exclusion [15].

Self-efficacy is the belief people have about their capabilities to successfully perform an outcome [16]. It is a fundamental coping mechanism and can be included in models of coping with, e.g., chronic illnesses or depressive symptoms [17]. For example, it has been shown that self-efficacy can moderate the relation between informal caregiving and depressive symptoms [18]. Similarly, we hypothesize that self-efficacy moderates the association between health comparisons and social exclusion, particularly in men. Because we assume that negative health comparisons are particularly important for men, we concentrated on the moderating role of self-efficacy among men. As outlined above, we hypothesize that individuals that are worse off than others might have feelings of embarrassment or shame. This might result in perceived social exclusion [15]. Individuals scoring high in self-efficacy might have better coping strategies to deal with negative health comparisons. Thus, it is conceivable that these individuals did not feel socially excluded because they might feel able to overcome these negative health comparisons (e.g., adopting a proactive approach). As self-efficacy is potentially modifiable [19], this knowledge might help to attenuate this association.

In sum, based on a population-based sample of community-residing individuals aged 40 and over, we aimed at investigating whether health comparisons are associated with social exclusion in the total sample and in both sexes among individuals in the second half of life. In addition, it was analyzed whether the relation between health comparisons and social exclusion is moderated by self-efficacy.

## Methods

### Sample

For the current study, data were derived from the German Ageing Survey (“Deutscher Alterssurvey”, DEAS) under the auspices of the German Federal Ministry for Family Affairs, Senior Citizens, Women, and Youth (BMFSFJ). It is funded by the BMFSFJ. The first wave of this survey was conducted in 1996. The German Centre of Gerontology (DZA) in Berlin is responsible for the DEAS study. Fieldwork was done by the Institute for Applied Social Sciences (infas). Main goals were to “provide a representative national database containing information describing the living conditions of the country’s middle-aged and older population and to study diversity within the older section of the population, the process of ageing as it affects individuals and processes of social change as they relate to old age and ageing” [20]. Thus, inclusion criteria was that individuals had to be 40 years and over. Thus, primary inclusion criterion was that individuals had to be 40 years and over. In further detail, inclusion criteria for first time participants were: (i) born between 1929 and 1974 and (ii) living in private household (excluding individuals living in institutionalized settings). Inclusion criteria for panel participants were: (i) at least one valid interview before (1996, 2002, 2008 or 2011), (ii) written consent (willingness to participate in the panel) given by baseline participants, (iii) still alive and not living abroad. The DEAS study has a cohort-sequential design, which means that it combines a large cross-sectional sample with longitudinal samples. A wide range of topics was covered in the DEAS study. For example, information on the household composition, attitudes, family structure, social networks, as well as physical and mental health were provided. Further details are provided elsewhere [20].

In the present study, cross-sectional data from the most recent wave (fifth wave: 2014) was used as social exclusion was exclusively quantified in this wave. In the fifth wave, 6002 individuals were interviewed and over 4000 respondents were re-interviewed. The response rate was 33%, which is comparable to other large survey studies performed in Germany [21]. In addition, this response rate mirrors the trend of decreasing participation rates in Western countries, particularly in Germany [22]. Efforts have been made to reduce this decrease in participation rates (e.g., increasing the incentives for respondents). Individuals were interviewed face-to-face by trained staff via computer-assisted personal interviewing (CAPI), covering, for example, sociodemographic variables. The average duration was about 96 min per CAPI interview. Subsequently, participants were asked to answer a standardized questionnaire that included topics such as social exclusion, self-efficacy or satisfaction with life. In total, *n* = 7838 filled out the questionnaire and provided information on social exclusion. Prior to the interview, written informed consent was given.

### Dependent variable

In the current study, social exclusion was measured using a scale constructed by Bude and Lantermann [23], consisting of four items (from 1 = “strongly agree” to 4 = “strongly disagree”): “I am worried to be left behind”, “I feel like I do not really belong to society”, “I feel that I am left out”, and “I feel excluded from society”. The scale was the average of at least two required (recoded) valid items, with higher values reflecting higher social exclusion In our study, Cronbach’s alpha equaled .88. While the original scale constructed by Bude and Lantermann [23] had six items, the short scale used in the present study had four items. This choice was made based on information from a pretest (*n* = 162). Thus, without losing reliability, two items (German language: “Ich werde ausgegrenzt”, „Ich habe das Gefühl, andere Menschen haben mich abgeschrieben“) were removed. This scale assesses the subjective feeling of social exclusion.

### Independent variables

Our independent variable of interest was the own health compared to others. It was measured with the item “How would you rate your health compared with other people your age” (much better; somewhat better; the same; somewhat worse, much worse).

According to Schwarzer and Jerusalem [24, 25], the degree of self-efficacy was measured, which consists of five items (ranging from 1 = “strongly agree” to 4 = “strongly disagree”): “It is easy for me to stick to my aims and accomplish my goals”, “I can usually handle whatever comes my way”, “I can solve most problems if I invest the necessary effort”, “If I am in trouble, I can usually think of a solution”, “When I am confronted with a problem, I can usually find several solutions”. The scale reflects the mean of at least 3 required valid items, all items have been recoded, with higher values reflecting higher self-efficacy Cronbach’s alpha was .84 in the current study. The self-efficacy scale was originally developed in German language. The original scale consists of ten items. In direct consultation with Ralf Schwarzer, the five-item version was made. It has been shown that this is a valid scale [26].

As potential confounders, we included age, marital status (married, living together with spouse; others (married, living separated from spouse; single; divorced; widowed)), region (West vs. East Germany), self-reported BMI (computed by self-reported weight (kg) divided by height-squared (meter), individual monthly net equivalent income (OECD scale), smoking status (daily smoker; casual smoker; former smoker; non-smoker), alcohol consumption and frequency of sports activities (categories were in both cases: ‘never’, ‘rarer than once a month’, ‘one to three times a month’, ‘once a week’, ‘several times a week’, and ‘daily’). In addition, the number of chronic conditions (e.g., bad circulation; cancer; diabetes; 0 to 11) and self-rated health status (from 1 = “very good” to 5 = “very bad”) were included as potential confounders.

### Statistical analysis

First, stratified by sex, sample characteristics are depicted. Second, adjusting for potential confounders, multiple linear regressions (total sample and stratified by sex) were conducted to test whether health comparisons and social exclusion are associated. Third, it was tested whether the association of health comparisons and social exclusion varies by degree of self-efficacy. To this end, an interaction term health comparisons x self-efficacy was included in the model. The statistical significance was determined with *p* < 0.05. Stata 14 was used for data analysis (StataCorp, College Station, TX, USA).

## Results

### Sample characteristics

An overview of the sample characteristics stratified by sex is presented in Table 1. While 76.7% of the male participants were married, and living together with spouse, 63.6% of the female participants were married, and living together with spouse. Average age was 65.5 years (SD: 11.2 years) in men, whereas it was 63.2 years (SD: 10.9 years) in women. Between men and women ratings of health comparisons were similar but statistically significantly different (Cramer’s V = .07, *p* < .001). Average social exclusion score was 1.6 (SD: 0.6 years) both in women and men.

**Table 1:** Sample characteristics stratified by sex (wave 5, *n* = 7838)

	Men (*n* = 3842)		Women (*n* = 3996)	
	N/Mean (Range)	%/(SD)	N/Mean (Range)	%/(SD)
Age in years	65.5 (40–93)	(11.2)	63.2 (40–95)	(10.9)
Marital status: married and living together with spouse’	2939	76.7%	2537	63.6%
Monthly net equivalent income in Euro	2018.6 (122–33,333)	(1497.0)	1870,7 (80–30,000)	(1256.1)
Region: West Germany	2598	67.6%	2668	66.7%
Body-Mass-Index (BMI)	27.4 (13.2–60.9)	(4.1)	26.4 (15.6–60.6)	(5.0)
Smoking status: Daily	539	14.1%	537	13.6%
- Yes, sometimes	176	4.6%	133	3.4%
- Not anymore	1745	45.7%	1134	28.8%
- Never been smoker	1362	35.6%	2138	54.2%
Consumption of alcohol: Daily	692	18.3%	233	5.9%
- several times a week	1184	31.2%	698	17.8%
- once a week	603	15.9%	641	16.3%
- one to three times a month	378	10.0%	560	14.2%
- less frequently	592	15.6%	1270	32.3%
- never	342	9.0%	532	13.5%
Physical activity: Daily	308	8.0%	355	8.9%
- several times a week	976	25.4%	1160	29.0%
- once a week	611	15.9%	816	20.4%
- one to three times a month	332	8.6%	260	6.5%
- less frequently	510	13.3%	408	10.2%
- never	1104	28.8%	997	25.0%
Number of physical illnesses	2.7 (0–11)	(1.9)	2.5 (0–10)	(1.9)
Self-rated health (from 1 = “very good” to 5 = “very bad”)	2.5 (1–5)	(0.8)	2.5 (1–5)	(0.8)
Health comparisons: Much better	532	14.1%	602	15.4%
Somewhat better	1648	43.7%	1469	37.5%
The same	1078	28.6%	1277	32.6%
Somewhat worse	397	10.5%	418	10.6%
Much worse	119	3.1%	154	3.9%
Self-efficacy (from 1 to 4; higher values reflect higher self-efficacy)	3.1 (1.2–4)	0.4	3.1 (1–4)	0.4
Social exclusion (from 1 to 4; higher values reflect higher social exclusion)	1.6 (1–4)	0.6	1.6 (1–4)	0.6

According to Schwarzer and Jerusalem [24, 25], self-efficacy was measured. Social exclusion was quantified using a scale constructed by Bude and Lantermann [23]

### Regression analysis

Results of regression analysis is described in Table 2. In the first column, the total sample was included, whereas in the second and third column exclusively men and women were included, respectively. In the fourth column, only men were included and an interaction term (self-efficacy x health comparisons) was integrated.

**Table 2:** Determinants of social exclusion (German Ageing Survey, 2014)

	(1)	(2)	(3)	(4)
	Social exclusion score – Total sample	Social exclusion score – Men	Social exclusion score - Women	Social exclusion score – Men; with interaction term (health comparisons x self-efficacy)
Potential confounders	✓	✓	✓	✓
Health comparison: much better (Ref.: The same)	−0.0394+	−0.0711*	−0.0153	−0.410*
	(0.0220)	(0.0307)	(0.0312)	(0.205)
Health comparison: somewhat better	−0.00447	−0.000603	−0.0118	−0.154
	(0.0162)	(0.0229)	(0.0228)	(0.177)
Health comparison: somewhat worse	0.0479+	0.0851*	0.0110	7.33e-06
	(0.0285)	(0.0393)	(0.0412)	(0.264)
Health comparison: much worse	0.138*	0.219**	0.0717	0.950**
	(0.0545)	(0.0833)	(0.0716)	(0.342)
Self-efficacy				−0.474***
				(0.0421)
Interaction term: Health comparison: much better x self-efficacy				0.124*
				(0.0625)
Interaction term: Health comparison: somewhat better x self-efficacy				0.0565
				(0.0560)
Interaction term: Health comparison: somewhat worse x self-efficacy				0.0224
				(0.0864)
Interaction term: Health comparison: much worse x self-efficacy				−0.264*
				(0.113)
Constant	2.643***	2.428***	2.829***	3.906***
	(0.0729)	(0.105)	(0.103)	(0.169)
Observations	6923	3472	3451	3467
R^2^	0.11	0.13	0.09	0.23

Comments: Potential confounders include age, employment status, family status, region, individual monthly net equivalent income, smoking status, body-mass-index, frequency of sports activities, alcohol consumption, self-rated health and number of chronic illnesses. Beta-Coefficients are reported; Cluster-robust standard errors in parentheses. *** *p* < 0.001, ** *p* < 0.01, * p < 0.05, + *p* < 0.10. Observations with missing values were dropped (listwise deletion). According to Schwarzer and Jerusalem [24, 25], self-efficacy was measured. Social exclusion was quantified using a scale constructed by Bude and Lantermann [23]

Adjusting for potential confounders, our regression analysis revealed that negative health comparisons were associated with feelings of social exclusion (‘much worse’, β = .14, *p* < .05; reference category: the same) in the total sample (first column). Positive health comparisons did not reach statistical significance in the total sample.

In men (second column), regression analysis showed that while positive health comparisons were slightly associated with social exclusion (‘much better’, β = −.07, *p* < .05), negative health comparisons were moderately associated with social exclusion (‘somewhat worse’, β = .09, p < .05; ‘much worse’, β = .22, *p* < .01). In women (third column), health comparisons were not associated with social exclusion.

In the fourth column, an interaction term self-efficacy x health comparison was included in the regression model for men. Findings revealed that the association between positive (‘much better’) as well as negative (‘much worse’) health comparisons and social exclusion were moderated by self-efficacy. However, it is worth noting that the association between health comparisons and social exclusion was not moderated by self-efficacy in the total sample and in women (results are not shown here, but are available upon request).

## Discussion

Based on a representative sample of community-residing individuals in the second half of life, the present study investigated whether health comparisons are associated with social exclusion in the total sample and in both sexes cross-sectionally. Main findings were that negative health comparisons are associated with feelings of social exclusion in men, but not women. Furthermore, positive health comparisons were weakly associated with decreased feelings of social exclusion in men. The association between negative as well as positive health comparisons and social exclusion was significantly moderated by self-efficacy in men.

It is conceivable that negative health comparisons are accompanied by feelings of exclusion from the society. It might be the case that men undertaking negative health comparisons cannot endure the thought that they are worse off than others. This might be explained by their competitive nature [14]. These feelings (e.g., feelings of shame, embarrassment or inferiority) might be strongly associated with perceived social exclusion [15].

Yet, studies are missing examining the association between health comparisons and social exclusion, which makes it difficult to compare our findings with previous studies. However, studies that used income comparisons instead demonstrated that these comparisons were mostly upwards. Furthermore, two recent longitudinal studies that also used data of the German Ageing Survey showed that changes to negative health comparisons are associated with changes in SWB and health. However, these two studies used other outcome measures. In sum, all these studies support the main idea (upwards health comparisons are associated with increased social exclusion) of the current study. Furthermore, it has been demonstrated that self-efficacy plays a role in this relationship in men.

We provide first evidence that negative health comparisons are associated with social exclusion. In the current study, data were derived from a population-based study. Social exclusion and self-efficacy were quantified using a scale with established psychometric properties. Adjusting for various potential confounders, our hypotheses are supported by the data even though alternative explanations of our findings are possible. This is a cross-sectional study, limiting the ability to draw causal conclusions. The causal direction might be reversed: Social exclusion might result in bad health and negative emotions, resulting in negative health comparisons. Sample selection bias is present in the German Ageing Survey. However, it has been proposed that this bias is rather small. Furthermore, the reference group for health comparisons was given in the questionnaire (health compared with other people of the same age). However, other factors (e.g., social networks) might also affect this process of health comparisons [27]. Nevertheless, we assume that age is the most salient dimension for comparing health.

## Conclusions

The findings of the present study suggests that negative health comparisons are associated with feelings of social exclusion in men. In conclusion, comparison effects are not symmetric and predominantly upwards among men in the second half of life. Strengthening self-efficacy might be helpful for attenuating this relationship.

## Abbreviations

BMFSFJ: Federal Ministry for Family Affairs, Senior Citizens, Women and Youth
BMI: Body-mass-index
DEAS: German Ageing Survey
DZA: German Centre of Gerontology
OECD: Organisation for Economic Co-operation and Development
SWB: Subjective well-being
